# Overlap of Promoter Recognition Specificity of Stress Response Sigma Factors SigD and SigH in *Corynebacterium glutamicum* ATCC 13032

**DOI:** 10.3389/fmicb.2018.03287

**Published:** 2019-01-09

**Authors:** Hana Dostálová, Tobias Busche, Jiří Holátko, Lenka Rucká, Václav Štěpánek, Ivan Barvík, Jan Nešvera, Jörn Kalinowski, Miroslav Pátek

**Affiliations:** ^1^Institute of Microbiology of the CAS, v. v. i., Prague, Czechia; ^2^Centrum für Biotechnologie, Universität Bielefeld, Bielefeld, Germany; ^3^Institute of Physics, Faculty of Mathematics and Physics, Charles University, Prague, Czechia

**Keywords:** *Corynebacterium glutamicum*, stress response, RNA-seq, *in vitro* transcription, sigma factor, promoter

## Abstract

*Corynebacterium glutamicum* ATCC 13032 harbors five sigma subunits of RNA polymerase belonging to Group IV, also called extracytoplasmic function (ECF) σ factors. These factors σ^C^, σ^D^, σ^E^, σ^H^, and σ^M^ are mostly involved in stress responses. The role of σ^D^ consists in the control of cell wall integrity. The σ^D^ regulon is involved in the synthesis of components of the mycomembrane which is part of the cell wall in *C. glutamicum*. RNA sequencing of the transcriptome from a strain overexpressing the *sigD* gene provided 29 potential σ^D^-controlled genes and enabled us to precisely localize their transcriptional start sites. Analysis of the respective promoters by both *in vitro* transcription and the *in vivo* two-plasmid assay confirmed that transcription of 11 of the tested genes is directly σ^D^-dependent. The key sequence elements of all these promoters were found to be identical or closely similar to the motifs -35 GTAAC^A^/_G_ and -10 GAT. Surprisingly, nearly all of these σ^D^-dependent promoters were also active to a much lower extent with σ^H^ *in vivo* and one (P*cg0607*) also *in vitro*, although the known highly conserved consensus sequence of the σ^H^-dependent promoters is different (-35 GGAA^T^/_C_ and -10 GTT). In addition to the activity of σ^H^ at the σ^D^-controlled promoters, we discovered separated or overlapping σ^A^- or σ^B^-regulated or σ^H^-regulated promoters within the upstream region of 8 genes of the σ^D^-regulon. We found that phenol in the cultivation medium acts as a stress factor inducing expression of some σ^D^-dependent genes. Computer modeling revealed that σ^H^ binds to the promoter DNA in a similar manner as σ^D^ to the analogous promoter elements. The homology models together with mutational analysis showed that the key amino acids, Ala 60 in σ^D^ and Lys 53 in σ^H^, bind to the second nucleotide within the respective -10 promoter elements (G**A**T and G**T**T, respectively). The presented data obtained by integrating *in vivo*, *in vitro* and *in silico* approaches demonstrate that most of the σ^D^-controlled genes also belong to the σ^H^-regulon and are also transcribed from the overlapping or closely located housekeeping (σ^A^-regulated) and/or general stress (σ^B^-regulated) promoters.

## Introduction

Most bacterial genes are transcribed by an RNA polymerase (RNAP) holoenzyme that includes a primary sigma subunit (σ factor) responsible for the transcription of housekeeping genes. In addition to the primary σ factor, a number of alternative sigma factors control various functions of the bacterial cells under specific nutritional, growth and environmental conditions. *Corynebacterium glutamicum* is a Gram-positive bacterium, which is applied in industrial biotechnology and also considered a model species for other corynebacteria as well as related actinobacterial genera such as *Mycobacterium* and *Rhodococcus*. The *C. glutamicum* genome encodes 7 σ factors: A primary σ^A^, a primary-like σ^B^ and five σ factors of Group IV (Gruber and Gross, 2003) also called extracytoplasmic function (ECF) σ factors (σ^C^, σ^D^, σ^E^, σ^H^, and σ^M^), which are involved in various stress responses.

Gene expression in bacteria is controlled by a complex regulatory network composed of protein transcription regulators, regulatory RNAs as well as low-molecular-weight metabolites. Sigma subunits of RNAP that are responsible for transcription initiation play an important role in this network. Groups of genes (σ factor regulons, called also sigmulons) controlled by different σ factors were found to constitute a modular network in *Pseudomonas aeruginosa* (Schulz et al., 2015). Individual separated sigmulons controlled by more than 25 σ factors display limited overlaps which probably allows this versatile bacterium to efficiently regulate cell processes and adapt to various environments as well as to the lifestyle of an opportunistic pathogen. The functions of the regulons of alternative σ factors in *P. aeruginosa* are significantly connected by the action of global transcriptional regulators which modulate transcription levels from the respective promoters (Binder et al., 2016). According to this hypothesis, modularity of regulatory networks may ensure rapid evolution (Binder et al., 2016). In contrast to previous notion concerning *P. aeruginosa*, considerable overlaps of σ factor regulons observed in many bacteria display regulatory redundancies and the ability to fine-tune responses to various environmental stimuli and combined stress conditions. Some genes belong to two or more sigmulons, since they are either transcribed from several promoters recognized by different alternative σ factors, or are transcribed with two or more different holoenzymes (RNAP core+σ) which drive transcription from the same promoter (Luo and Helmann, 2009). Microarray analysis revealed extensive coregulation of the genes involved in many cell processes, which are co-operatively controlled by four alternative σ factors (σ^B^, σ^C^, σ^H^, and σ^L^) in *Listeria monocytogenes* (Chaturongakul et al., 2011). Significant regulatory overlaps between the target genes of three σ factors (σ^M^, σ^W^, and σ^X^) involved in cell surface stress responses were also found in *Bacillus subtilis* (Jordan et al., 2008). A single promoter of the *sigB* gene encoding an alternative σ in *Mycobacterium tuberculosis* was recognized by as many as three different ECF σ factors (σ^E^, σ^H^, and σ^L^) (Dainese et al., 2006). Overlapping σ factor specificity in promoter recognition can be therefore considered to be a common feature of regulatory networks in bacteria.

Various types of stress conditions which bacteria encounter in the environment (e.g., heat, cell surface and oxidative stresses) often result in damaged proteins, which are then degraded by proteases or re-folded by chaperones during the stress response. Expression of some genes encoding proteases (*clpB*) and chaperones (*dnaK* and *dnaJ2*) was found to be activated by both σ^H^ (involved in heat and oxidative stress response) and σ^E^ (involved in cell surface stress response) in *C. glutamicum* (Šilar et al., 2016). The *clgR* gene encoding a heat-stress-responsive regulator was also found to be σ^H^ and σ^E^-dependent (Šilar et al., 2016). ClgR controls expression of several genes involved in protein quality control, and the function of ECF σ factors and the transcriptional regulator is thus integrated into a network. The same σ^H^/σ^E^ overlap was found in the transcription initiation of the *C. glutamicum sigB* gene, encoding the general stress response sigma factor σ^B^ (Dostálová et al., 2017). The possible activity of both σ^H^ and σ^M^ (involved in oxidative stress response) at the same *C. glutamicum* promoters was suggested, but has not yet been proven (Nakunst et al., 2007; Šilar et al., 2016; Dostálová et al., 2017), although the respective promoter consensus sequences seem very similar. Promoters of σ^C^-regulated genes in *C. glutamicum* were found to be different from other promoters recognized by ECF σ factors, and σ^C^ regulon, which includes genes involved in the aerobic respiratory chain in *C. glutamicum*, seems to be separated from the other σ factor regulons (Toyoda and Inui, 2016).

Many stress response genes of *C. glutamicum* were found to be transcribed not only from stress promoters, but also from overlapping or closely located housekeeping promoters controlled by σ^A^ and/or σ^B^ (Engels et al., 2004; Ehira et al., 2009; Busche et al., 2012; Šilar et al., 2016). Such transcription of stress-response genes from multiple promoters also seems to be frequent in other bacteria (Seo et al., 2012; Cho et al., 2014). The activity of σ^A^ and/or σ^B^ under unstressed conditions probably ensures a basal level of expression of the respective genes which also play important roles in fast-growing bacterial cells.

We have recently found by using RNA-seq that σ^D^-dependent genes are involved in the synthesis of components of the mycomembrane, which is a part of the cell wall in *C. glutamicum* ATCC 13032. The σ^D^ regulon, including 29 genes, thus contributes to the maintenance of cell wall integrity (Taniguchi et al., 2017). Similar group of σ^D^-dependent genes was also detected in the strain *C. glutamicum* R using different techniques (Toyoda and Inui, 2018). In *C. glutamicum* R, the *sigD* overexpression conferred lysozyme resistance and the *lppS* gene (encoding L,D-transpeptidase) and probably some other σ^D^-dependent genes were found to contribute to lysozyme resistance. No clear σ^D^ regulon induction was observed in this strain under any other stress conditions tested (treatment with sodium dodecyl sulfate, cetyl trimethylammonium bromide, ethambutol or ampicillin) (Toyoda and Inui, 2018).

Phenol was previously described to cause a significant oxidative stress at subtoxic concentrations. At higher concentrations, phenol affects bacterial cells by releasing cell wall components and causing non-specific increase in cell permeability (Denyer, 1995). At the level of the cytoplasmic membrane, phenol induces a loss of structural integrity by the leakage of potassium ions and various organic compounds and can also displace phospholipid molecules in the cell envelope (Denyer, 1995). Such damage to the cell wall might induce a similar stress response as lysozyme treatment. However, effects of toxic aromatic compounds, such as phenol, vanillin and ferulic acid, which are degraded by *C. glutamicum* (Chen et al., 2016, 2017, 2018), on σ^D^ regulon induction have not yet been tested.

Although the number of precisely localized promoters driving expression of σ^D^-dependent genes was not high, it was possible to propose their consensus sequence. Considering the difference between σ^D^-controlled promoters and other promoter classes, the σ^D^ regulon seemed to also be an insulated gene group.

In this study, we discovered that nearly all of the σ^D^-dependent promoters found in *C. glutamicum* ATCC 13032 are also to a much lower extent active with RNAP+σ^H^, although the known highly conserved consensus sequence of the σ^H^-dependent promoters differs from that of the σ^D^-dependent promoters. We have found that phenol in the cultivation medium acts as a stress factor, inducing the expression of some σ^D^-dependent genes. To achieve reliable results, we combined the genome-wide technique (RNA-seq) and single-promoter analysis (*in vitro* transcription and an *in vivo* two-plasmid system for the assignment of σ factors to genes/promoters). *In silico* homology modeling completed the different approaches to analyzing the sigma-promoter relationships.

## Materials and Methods

### Bacterial Strains, Plasmids, Oligonucleotides and Growth Conditions

*Escherichia coli* DH5α (Hanahan, 1985) was cultivated aerobically in 500-ml flasks containing 70–100 ml of 2xYT medium (Green and Sambrook, 2012) at 37°C. Wild-type (WT) *C. glutamicum* ATCC 13032, its deletion derivative *C. glutamicum* Δ*sigD* (Taniguchi et al., 2017) and *C. glutamicum sigD*-overexpressing strain (Taniguchi et al., 2017) were used for DNA and RNA isolations, and as hosts for testing the activities of promoters cloned in the promoter-test vector pEPR1. *C. glutamicum* was cultivated aerobically in 500-ml flasks with 70–100 ml of complete 2xYT medium or in minimal CGXII medium (Keilhauer et al., 1993) at 30°C. Media were supplemented with antibiotics, when necessary: kanamycin (Km; 30 μg/ml), tetracycline (Tc; 10 μg/ml) or ampicillin (Ap; 100 μg/ml). The plasmid vectors used are listed in Table 1. The oligonucleotides used are listed in Supplementary Table S1.

**Table 1:** Plasmid vectors used in this study.

Plasmid	Characteristics	Source
pRLG770	*E. coli* vector, *rrnB* terminator, Ap^R^, used for *in vitro* transcription analysis	Ross et al., 1990
pEPR1	*E. coli-C. glutamicum* promoter-test vector, Km^R^, promoter-less *gfpuv* as a reporter	Knoppová et al., 2007
pEC-XT99A	*E. coli-C. glutamicum* expression vector, Tc^R^, IPTG-inducible *trc* promoter	Kirchner and Tauch, 2003


### DNA Manipulations

DNA isolation, PCR, cutting by restriction enzymes, ligation and transformation of *E. coli* were done using the standard techniques (Green and Sambrook, 2012). *C. glutamicum* cells were transformed by electroporation (van der Rest et al., 1999). DNA fragments for cloning promoters in pRLG770 and pEPR1 were assembled from the synthetized complementary oligonucleotides of around 75 nt in length, with overhangs ready for ligation. Their sequences are shown in Supplementary Table S1. Mutations in *sigH* were constructed using a Q5^®^ Site-Directed Mutagenesis Kit (New England BioLabs^®^ Inc.) according to the recommendations of the manufacturer. The specific oligonucleotide primers for mutagenesis were designed by NEBaseChanger^TM^ v1.2.8 (New England BioLabs^®^ Inc.).

### RNA Isolation, cDNA Library Preparation and Sequencing

*Corynebacterium glutamicum* ATCC 13032 was cultivated in minimal medium CGXII with glucose (2%) or phenol (3.4 mM). The *sigD-*overexpressing *C. glutamicum* strain was used as described previously (Taniguchi et al., 2017). Total RNA was isolated from 3 biological replicates of *C. glutamicum* cells grown to the exponential phase using a Quick-RNA Miniprep Plus kit according to the manufacturer’s instructions (Zymo Research). After additional DNase treatment, RNA samples were purified with an RNA Clean&Concentrator-5 kit (Zymo Research) and quantified with a DropSense 16 (Trinean). The quality of total RNA was controlled with an RNA 6000 Nano kit in an Agilent 2100 Bioanalyzer (Agilent Technologies). To construct the whole transcriptome cDNA library, 2.5 μg total RNA (RIN > 9) was used for the depletion of rRNA with a Ribo-Zero rRNA Removal Kit (Bacteria) according to manufacturer’s instructions (Illumina). The rRNA removal was checked with an Agilent RNA Pico 6000 kit and the Agilent 2100 Bioanalyzer (Agilent Technologies). The mRNA obtained was converted to a cDNA library according to the TruSeq Stranded mRNA Sample Preparation guide (Illumina). The quality and quantity of the cDNA library was checked with an Agilent High Sensitivity DNA kit and the Agilent 2100 Bioanalyzer (Agilent Technologies). Sequencing was performed on an Illumina HiSeq 1500 using 70 bases read length (Illumina).

A primary 5′-end specific cDNA library was synthesized using 2 × 2.5 μg total RNA (RIN > 9) as described previously (Kranz et al., 2018). Briefly, after rRNA depletion and quality control, as described for the whole transcriptome cDNA libraries, a terminator 5′-phosphate-dependent exonuclease treatment (TEN, Illumina) was carried out to digest non-primary transcripts. The remaining non-digested non-primary transcripts were tagged by the ligation of the RNA 5′-index adapter 5′-CCCUACACGACGCUCUUCCGAUCGAG-**UACCCUAG** (index in bold) to the 5′-monophosphorylated ends. The primary 5′-triphosphate ends were converted to 5′-mono-phosphate by RNA 5′-polyphosphatase (RPP) treatment (Epicenter) to ligate the 5′-adapter to the 5′-ends of primary transcripts. Finally, reverse transcription with a stem-loop DNA adapter and library amplification was performed. The quality and quantity of the cDNA library was checked in the same way as for the whole transcriptome cDNA library. Prior to sequencing, primary 5′-end cDNA libraries were purified and size-selected for fragments approximately 100–1000 bases long via gel electrophoresis, and quantified again. Paired end sequencing was performed in an Illumina MiSeq using MiSeq Reagent Kits v3 with a read length of 2 × 75 bases (Illumina).

### Read Processing, Mapping and Visualization

Paired-end reads were mapped to the *C. glutamicum* ATCC 13032 reference genome sequence accession number BX927147 (Kalinowski et al., 2003) with bowtie2 v2.2.7 (Langmead and Salzberg, 2012) using the default settings for paired-end read mapping.

False-positive primary transcriptome cDNA reads containing the barcode sequence TACCCTAG at their 5′-ends were discarded. The remaining R1 cDNA reads were mapped to the *C. glutamicum* ATCC 13032 reference genome sequence accession number BX927147 (Kalinowski et al., 2003) with bowtie2 v2.2.7 (Langmead and Salzberg, 2012) using the default settings for single-end read mapping. Read Explorer v.2.2 (Hilker et al., 2014) was used for the visualization of short read alignments, transcription start site (TSS) detection and differential gene expression analysis.

### Identification of Transcription Start Sites (TSS)

Transcription start sites (TSS) were detected essentially as described by Wittchen et al. (2018). Primary 5′-end cDNA library data were analyzed with the software ReadXplorer v2.2 (Hilker et al., 2016) using the *Transcription analysis* function. The parameters for TSS detection were chosen by ReadXplorer using its automatic parameter estimation. TSS were detected with at least 10 read starts and a minimal coverage increase of 100%. The resulting list of predicted TSS was manually checked for false-positives.

### Differential Gene Expression Analysis

Differential gene expression analysis of *C. glutamicum* grown with and without phenol (3.4 mM), including normalization, was performed using the whole transcriptome data and Bioconductor package DESeq2 (Love et al., 2014) included in the software ReadXplorer v2.2 (Hilker et al., 2016). The signal intensity value (*a*-value) was calculated by the log2 mean of normalized read counts, and the signal intensity ratio (*m*-value) by log2 fold-change. The evaluation of the differential RNA-seq data was performed using an adjusted *p*-value cut-off of *P* ≤ 0.01 and a signal intensity ratio (*m*-value) cut-off of ≥1 or ≤-1.

### *In vitro* Transcription

The *in vitro* transcription assay was carried out essentially as described previously (Holátko et al., 2012). The holo-RNAP was reconstituted from the RNAP core enzyme isolated from *C. glutamicum* and individual *C. glutamicum* sigma factors isolated as His-tagged recombinant proteins from *E. coli*. The RNAP core (100 nM) was mixed with the respective σ factor (σ^C^, σ^D^, σ^E^, σ^H^, or σ^M^) in a molar ratio of 1:30. The holo-RNAP was assembled for 10 min at 37°C. The transcription mixture was incubated for 15 min at 37°C. The transcripts labeled with [α-^32^P]UTP were separated in 5.5% polyacrylamide gel. *In vitro* transcription assays were done 2 or 3 times for each promoter, with essentially the same results.

### Promoter Activity Measurements Using the Two-Plasmid System

Sigma factors were assigned to individual promoters *in vivo* using the two-plasmid system for *C. glutamicum* described recently (Dostálová et al., 2017) and similar that developed for the identification of σ^E^-dependent promoters in *E. coli* (Rezuchova and Kormanec, 2001). In principle, the sigma factors overproduced using the expression vector pEC-XT99A drove transcription from the individual promoters transcriptionally fused to the *gfpuv* reporter gene in the other plasmid present in the cell, the promoter-test vector pEPR1. Promoters were cloned to pEPR1 as approximately 75-nt fragments using the restriction sites *Pst*I and *Bam*HI. The same pEC-XT99A constructs as described previously (Dostálová et al., 2017) were used to overexpress the *sig* genes after the addition of isopropyl-β-thiogalactopyranoside (IPTG). Two-plasmid strains were cultivated in 2xYT medium (containing Km and Tc) for 24 h and the cell samples were collected. The cells were disrupted with a FastPrep homogenizer (MP Biomedicals). The fluorescence of the cell-free extracts was determined with a Saphire2 microplate spectrophotometer (Tecan; excitation wavelength 397 nm; emission wavelength 509 nm). Cells harboring the pEPR1 construct and the expression vector without a *sig* gene were used as a control. To determine the background fluorescence of *C. glutamicum* cells, the strain only carrying the promoter-less vector pEPR1 was used. Protein concentration in the extract was determined by Bradford assay, and promoter activity was expressed in arbitrary units/mg protein.

### Homology Modeling and Molecular Dynamics (MD) Simulations

The homology models of the σ^D^ and σ^H^ domains which recognize the -10 and -35 sequences of the respective promoters were produced by using the Swiss-Model server (Waterhouse et al., 2018). The crystal structures of *E. coli* σ^E^, PDBid: 4LUP (for -10 element GTC) (Campagne et al., 2014) and PDBid: 2H27 (for -35 element GGAAC) (Lane and Darst, 2006) were used as templates. The nucleotides within the *E. coli* σ^E^ consensus were replaced to match the consensus for *C. glutamicum* σ^H^ or σ^D^, where necessary. Molecular dynamics simulations were done using the software package AMBER (Salomon-Ferrer et al., 2013) and Linux computer nodes with powerful NVIDIA GPUs that enable the accumulation of 50-ns MD trajectories at 280 K.

## Results

### Mapping of Transcription Start Sites of σ^D^-Dependent Genes by RNA-seq

RNA-seq with a *C. glutamicum* WT strain, a strain overexpressing *sigD* and a *sigD* deletion (Δ*sigD*) strain was performed previously (Taniguchi et al., 2017). Using this approach, the 29 genes were identified, which showed the increased transcription in *C. glutamicum* overexpressing *sigD* and decreased transcription in the Δ*sigD* strain. The genes selected may be those which are directly under the control of σ^D^, or the genes whose transcription is influenced by an indirect effect of *sigD* overexpression or deletion.

To further investigate these σ^D^-dependent genes and to map their transcriptional start sites (TSS), RNA-seq of 5′-enriched primary transcripts of *C. glutamicum* overexpressing *sigD* was used. The sequencing of primary 5′-end specific cDNA library allows the exact mapping of TSS. To detect these TSS, the data obtained from the primary 5′-end cDNA sequencing were mapped to *C. glutamicum* ATCC 13032 reference genome sequence, accession number BX927147 (Kalinowski et al., 2003) and analyzed via ReadXplorer (Hilker et al., 2016). To distinguish between the background and actual TSS signal, we considered a position to be +1 (TSS) if the number of read starts here was 10 times higher than at position -1. In this way the 5′- ends of the transcripts of the above mentioned 29 putative σ^D^-dependent genes were detected. Since several genes were cotranscribed in operons, the total number of TSS was 23 (Supplementary Table S2). The 50-nt sequences (-49 to +1) were aligned at the TSS, and the consensus motifs within the region of the supposed promoters were searched using the software Improbizer (Ao et al., 2004) as described previously (Albersmeier et al., 2017). The consensus sequence or closely similar motifs within the -35 and -10 regions were found in 11 sequences (Table 2). The products of the respective genes are involved in the synthesis of corynomycolic acid (*fadD2*, *cmt1*, *cmt2*, *cmt3*), peptidoglycan formation (*lppS*), inhibition of σ^D^ activity (*rsdA*) or have hypothetical functions (Table 3). The σ^D^ is thus involved in the control of cell wall integrity and cell envelope stress response, as described previously in the strains *C. glutamicum* ATCC 13032 (Taniguchi et al., 2017) and *C. glutamicum* R (Toyoda and Inui, 2018).

**Table 2:** Promoter sequences of σ^D^-dependent genes in *C. glutamicum* ATCC 13032 determined by RNA sequencing specific for transcription start sites.

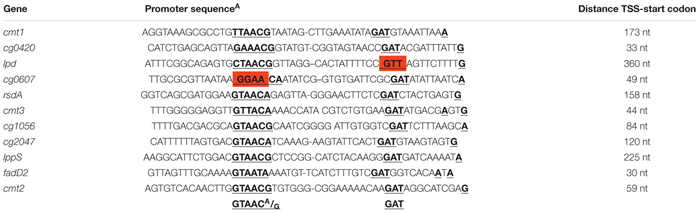

*^*A*^The predicted -35 motif GTAAC****^*A*^/_*G*_****and -10 motif GAT are in bold, underlined. Elements identical to the consensus of σ^*H*^-controlled promoters are highlighted in red. The gaps were introduced to align the key motifs*.

**Table 3:** Products of σ^D^-dependent genes in *C. glutamicum* ATCC 13032.

Gene ID^A^	Gene name	Function
*cg0413*	*cmt1*	Trehalose corynomycolyl transferase
*cg0420*		Hypothetical glycosyltransferase
*cg0441*	*lpd*	Dihydrolipoamide dehydrogenase
*cg0607*		Hypothetical secreted protein
*cg0697*	*rsdA*	Anti-sigma D
*cg1052*	*cmt3*	Trehalose corynomycolyl transferase
*cg1056*		Hypothetical membrane protein
*cg2047*		Hypothetical secreted protein
*cg2720*	*lppS*	L,D-transpeptidase
*cg3179*	*fadD2*	Long-chain fatty-acid-CoA ligase
*cg3186*	*cmt2*	Trehalose corynomycolyl transferase


*^*A*^Genes are listed according to CoryneRegNet (http://www.coryneregnet.de/).*

**FIGURE 1:**
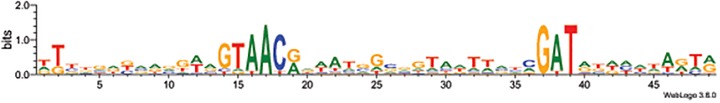
Sequence logo of the 11 studied σ^D^-dependent promoters. The logo was made using Weblogo3 (Crooks et al., 2004).

The sequences were used to generate a sequence logo with Weblogo3 (Figure 1). The consensus sequences GTAAC**^A^/_G_** as the -35 motif and GAT as the -10 motif are apparent.

### *In vitro* Transcription From the σ^D^-Controlled Promoters

**FIGURE 2:**
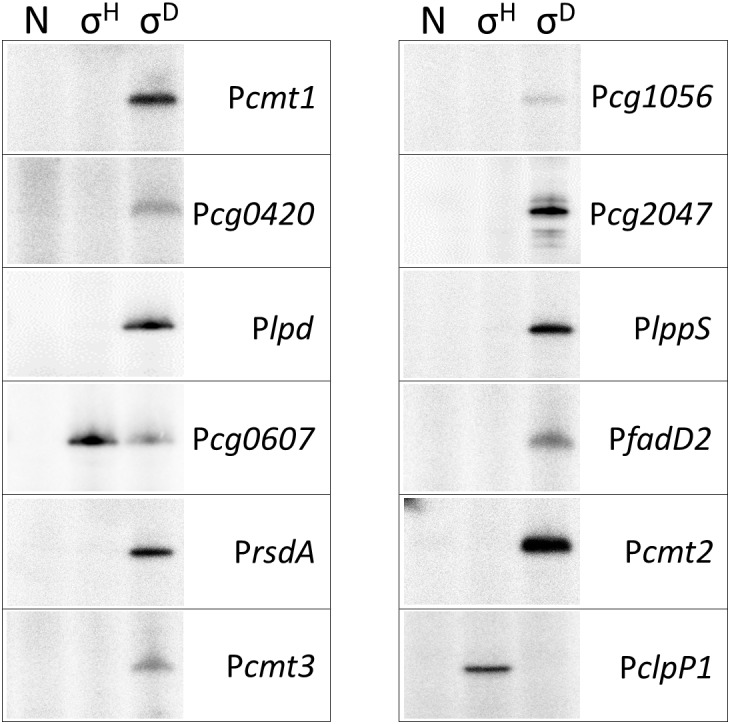
*In vitro* transcription driven from σ^D^-dependent promoters with reconstituted *C. glutamicum* holo-RNAPs (autoradiogram of the SDS-PAGE gel). The lanes with no sigma (N), σ^H^ and σ^D^ are shown. *In vitro* transcription was carried out as described previously (Holátko et al., 2012). The derivatives of the plasmid vector pRLG770 with inserted promoter fragments (approximately 75 nt) were used as DNA templates, recombinant RNAP core was isolated from *C. glutamicum*, and sigma factors were isolated as His-tagged proteins from *E. coli* using the pET-22b(+) constructs carrying the respective inserted *sig* genes (Holátko et al., 2012; Šilar et al., 2016). The σ^H^-specific promoter P*clpP1* was used as a control. Representative results of 2–3 assays are shown.

The activity of the 11 proposed σ^D^-dependent promoters, which were deduced from the TSS determination by RNA-seq, was tested using the *in vitro* transcription system (Holátko et al., 2012). The promoter sequence elements of the proposed σ^D^-dependent promoters were apparently different from the consensus sequence of σ^A^/σ^B^-driven promoters (Pátek and Nešvera, 2011; Albersmeier et al., 2017) and therefore ECF σ factors (σ^C^, σ^D^, σ^E^, σ^H^, and σ^M^) were only used. This assay may directly prove whether a promoter is specifically recognized by RNAP associated with the chosen σ factor, and thus distinguish between direct and indirect control by the tested σ. As shown in Figure 2, all but one promoter were found to be exclusively σ^D^-specific. The only exception was the promoter of *cg0607* (P*cg0607*), which was active with both σ^D^ and σ^H^. The *in vitro* transcript level with σ^H^ was even higher than with σ^D^ (Figure 2). It is worth mentioning that P*cg0607* is the only one out of the 11 analyzed promoters that has the -35 sequence element GGAAC which is a consensus for σ^H^-dependent promoters (Busche et al., 2012). The ECF σ factors σ^C^, σ^E^, and σ^M^ did not provide any signal in the assays (data not shown). The σ^H^-specific *clpP1* promoter (Busche et al., 2012) was used as a control.

### Determination of Activity of σ^D^-Controlled Promoters *in vivo* Using Two-Plasmid System

**FIGURE 3:**
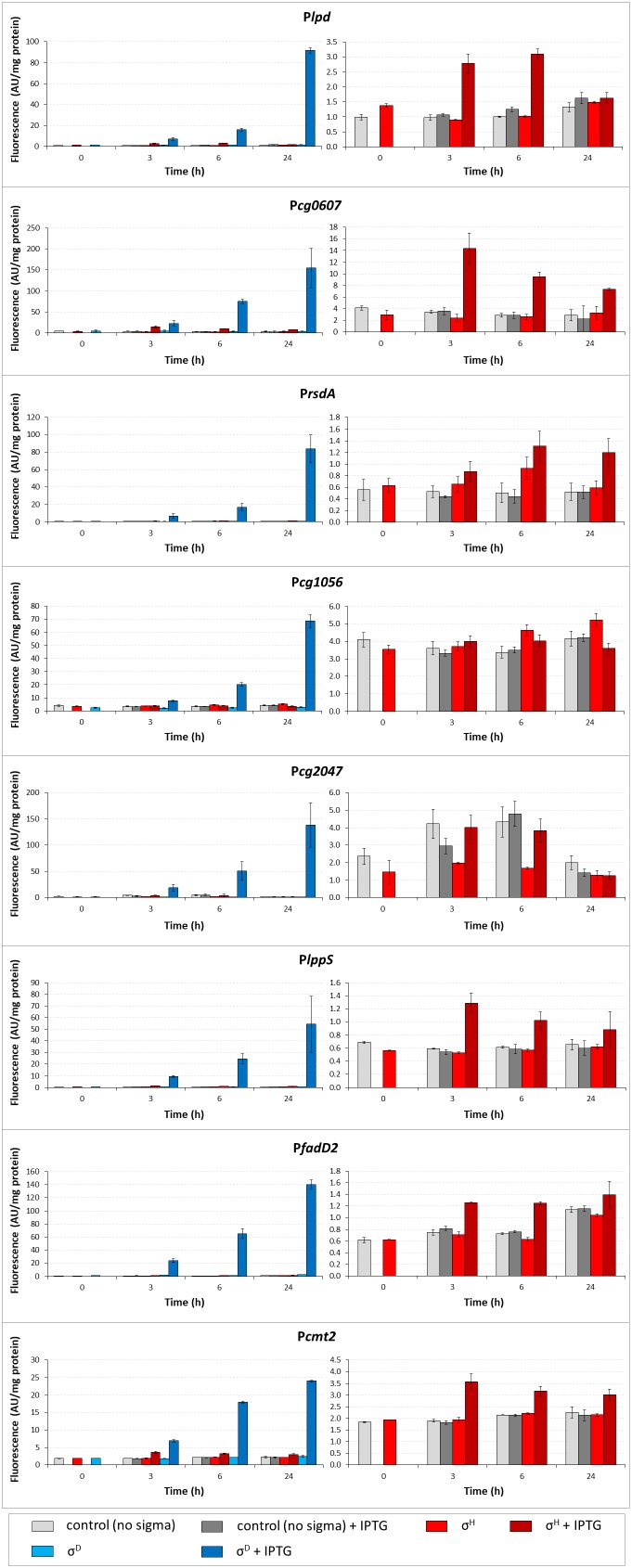
Determination of promoter activity under *sigD* or *sigH* overexpression conditions using the two-plasmid assay. Promoter activity was measured as Gfpuv fluorescence intensity of cell extracts shown as bars in colors representing respective sigma factors. The *C. glutamicum* strains carried the pEC-XT99A constructs overexpressing *sigD* (blue bars) or *sigH* (red bars) after IPTG addition (added at time point 0, dark bars) and the promoter-test vector pEPR1 carrying the reporter *gfpuv* gene downstream of the target promoter (P*lpd*, P*cg0607* P*rsdA*, P*cg1056*, P*cg2047*, P*lppS*, P*fadD2*, or P*cmt2*). The strains harboring the pEPR1 with the target promoters and empty pEC-XT99A were used as the controls (gray bars). Only the σ^H^-induced activity and controls are shown in the right panels to emphasize the minor recognition of σ^D^-controlled promoters by σ^H^. The background fluorescence intensity with control cells only carrying the empty vector pEPR1 was 0.25 ± 0.09 in all cases. AU, arbitrary units. The standard deviations of three biological replicates are depicted by error bars.

To confirm the results of *in vitro* transcription, the activity of σ^D^-dependent promoters was determined *in vivo* using the two-plasmid *C. glutamicum* cells carrying a *sig* gene in the expression vector pEC-XT99A and the analyzed promoter in the promoter-test vector pEPR1 (Dostálová et al., 2017). The promoter activity was measured as the fluorescence intensity of the Gfp reporter protein. The results of the assays with 7 most felicitous example promoters which confirmed the activity of σ^H^ in transcription are presented in Figure 3. As shown in the left part of Figure 3, all promoters exhibited high activity with the overexpressed *sigD*. Results with P*cg1056* were included to document that not all σ^D^-controlled promoters are also recognized by σ^H^, although there is no apparent reason found by inspection of the promoter sequence only. Activity of P*cg2047* with σ^H^ was approximately twice higher after induction at time point 3 (T3) and time point 6 (T6) although it was at the same level of the control. It is an unusual feature of this two-plasmid system in some cases, which we described in our previous paper (Dostálová et al., 2017). We concluded, therefore, that there was an activity of P*cg2047* initiated by σ^H^.

The level of Gfp reporter fluorescence was lowest 3 h (T3) after the addition of the inducer (IPTG) in all cases and highest after 24 h (T24) in 10 of the 11 tested promoters. The promoters P*cmt2*, P*fadD2*, P*rsdA*, P*cg2047*, P*lppS*, P*lpd*, and P*cg0607* were also found to be active with σ^H^, as shown in the right part of Figure 3. Although the maximal σ^H^-induced activities were 1.5- to 64-fold lower than those with σ^D^ under the conditions used, the effect was apparent (Figure 3, right part). The promoter P*cg1056* is an example of a promoter which did not demonstrate any increase in activity with *sigH* overexpression. Neither of the other overproduced σ factors supported a promoter activity increase with any of the promoters tested (data not shown). Interestingly, the trend of changes in the σ^H^-directed promoter activity was mostly the opposite of that with σ^D^: the activity with σ^H^ was highest 3 h after the addition of IPTG, then mostly decreased at T6, and the difference in Gfp level between the cells with IPTG-induced and non-induced σ^H^-overproduction nearly vanished at T24 in most cases (Figure 3, right part). Due to this trend, σ^D^- and σ^H^-incited promoter activities were nearly comparable at T3. This effect was most apparent in P*cg0607*, which was also found to be active with σ^H^ by *in vitro* assay (Figure 2): this ratio between promoter activity with σ^D^/σ^H^ was 1.5 at T3 but 20.5 at T24 (Figure 3). The overall conclusion is that most of the σ^D^-controlled promoters are also to a lesser extent σ^H^-activated.

### Promoter Activity in the *sigD* Deletion Strain (Δ*sigD*)

**FIGURE 4:**
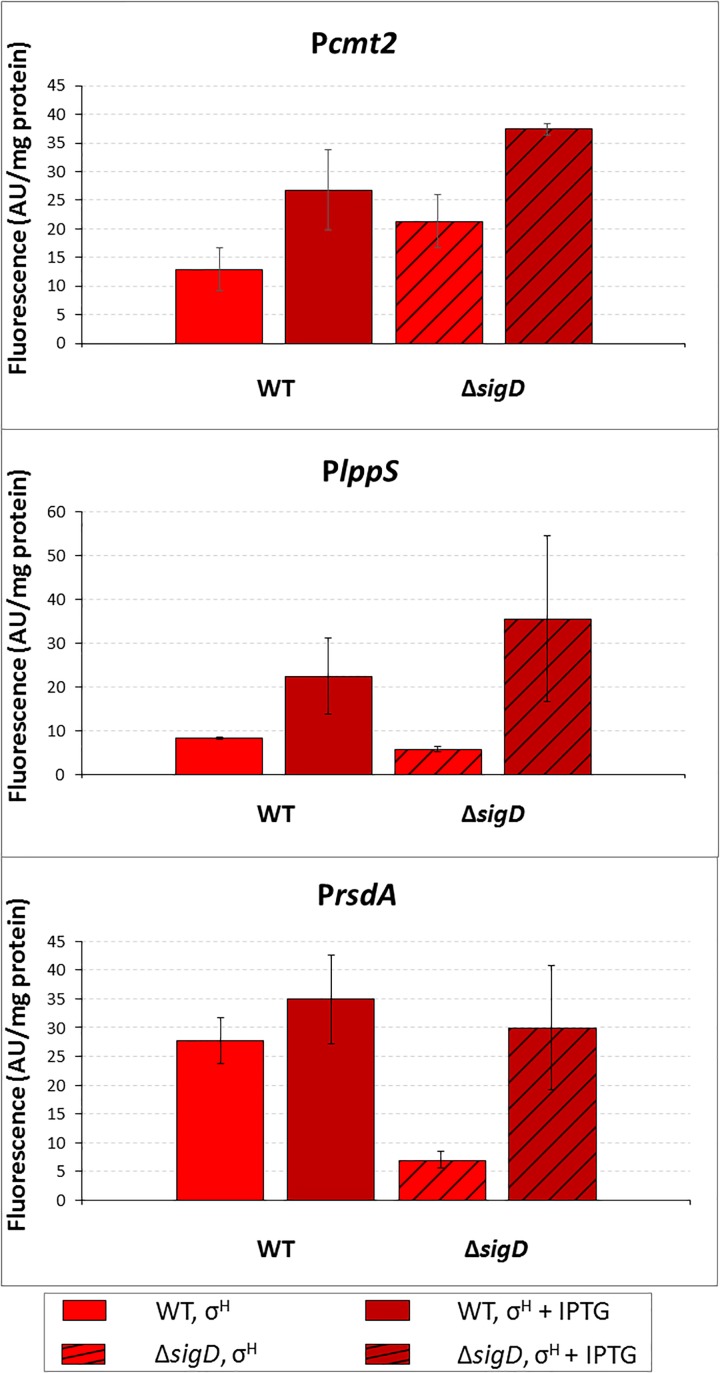
Determination of promoter activity in *C. glutamicum* WT and Δ*sigD* strains under *sigH* overexpression conditions using the two-plasmid assay. The *C. glutamicum* strains carried the vector pEC-XT99A construct overexpressing *sigH* with IPTG and the promoter-test vector pEPR1 with a target promoter P*cmt2*, P*lppS* or P*rsdA*. The promoter activity was measured as the Gfpuv fluorescence intensity of the cell extracts after 6-h growth on complete agar plates with or without IPTG. Fluorescence of cultures without IPTG are shown as light bars; fluorescence of cultures with IPTG induction are shown as dark bars. Open bars depict fluorescence of the strains with WT background, hatched bars depict fluorescence of the strains with the Δ*sigD* background. AU, arbitrary units. The standard deviations of three biological replicates are depicted by error bars.

To prove that σ^H^ is directly responsible for the minor activity of σ^D^-controlled promoters *in vivo*, we tried to overexpress *sigH* in the Δ*sigD* strain also harboring pEPR1 with the target promoters. The Δ*sigD* strain grew slightly slower than the wild type strain (Taniguchi et al., 2017). However, the Δ*sigD* clones carrying two vector constructs grew extremely poorly. Therefore, we only succeeded in testing the clones with *Pcmt2*, *PlppS*, and *PrsdA* (cloned in pEPR1), moreover, only on a solid agar medium. The increase in promoter activity, (induction ratio of WT and Δ*sigD* strains with *sigH* overexpressed), was 2.1 and 1.8, respectively, for P*cmt2*, 2.2 and 3.5, respectively, for P*lppS* and 1.1 and 6.1, respectively, for P*rsdA* (Figure 4). Thus, RNAP+ σ^H^ was apparently directly active in transcription from all three tested σ^D^-dependent promoters.

### Analysis of *C. glutamicum* σ^D^ and σ^H^ Protein Sequence Similarity

**FIGURE 5:**
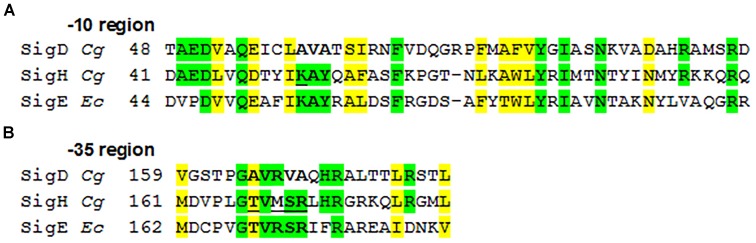
Alignment of the σ AA sequences binding promoter –35 and –10 elements in *C. glutamicum*. *E. coli* σ^E^, which was analyzed using its crystal structure (Lane and Darst, 2006; Campagne et al., 2014), is aligned for comparison. The AAs which are supposed to interact with the –10 **(A)** and –35 **(B)** elements (Lane and Darst, 2006; Campagne et al., 2014) are in bold. The σ^H^ AAs which we replaced with the corresponding AAs from σ^D^ by mutagenesis are underlined. Identical AAs are shown in green, similar in yellow; *Cg*, *C. glutamicum*; *Ec*, *E. coli*.

To explain the observed function of σ^H^ in transcription from some σ^D^-dependent promoters, the similarity of the amino acid sequences of *C. glutamicum* σ^D^ and σ^H^ proteins was analyzed. The level of overall similarity of the σ^D^ and σ^H^ sequences is very low (<25% identical AA), in contrast to *C. glutamicum* ECF sigma factors σ^H^, σ^E^, and σ^M^, which exhibit a 25–38% mutual identity of AAs. However, when the regions which are thought to interact with the -35 and -10 promoter sequences (Lane and Darst, 2006; Campagne et al., 2014) were compared, some AAs were found to be identical or similar in σ^D^ and σ^H^ (shown in green and yellow, respectively, in Figure 5). As shown in Figure 5, majority of these AAs were also found to be conserved in the model ECF sigma factor, σ^E^ from *E. coli*, whose crystal structure was previously used for studies on sigma-promoter DNA interactions (Lane and Darst, 2006; Campagne et al., 2014).

### Computer Modeling of σ^D^ and σ^H^ in Complexes With -10 and -35 Elements of σ^D^- and σ^H^-Controlled Promoters

We have shown that the consensus sequences of the *C. glutamicum* -10 and -35 elements recognized by σ^D^ (this study) and σ^H^ (Busche et al., 2012) differ (i.e., G**A**T vs. G**T**T at -10 and G**T**AA**C**^A^/_G_ vs. G**G**AA**^T^**/_C_ at -35). To identify the key AAs responsible for recognizing different nucleotides at the second position of these consensus sequences and to understand why σ^H^ is, to a certain extent, able to initiate transcription from the σ^D^-dependent promoters, we used computer modeling tools. First, we created homology models of those σ^D^ and σ^H^ domains, which recognize the -10 and -35 sequences of the respective promoters. The structure of *E. coli* σ^E^ based on the crystal protein in complex with promoter DNA was used as a template (Lane and Darst, 2006; Campagne et al., 2014). It should be noted that the DNA promoter sequences recognized by *E. coli* σ^E^ are closer to the *C. glutamicum* consensus of the σ^H^-specific -10 promoter element (GTC in *E. coli* σ^E^, i.e., T at the second position in both cases) as well as the -35 element (GGAAC in *E. coli* σ^E^, i.e., G in the second position in both cases). This also corresponds to the similarity of the AA sequences of *E. coli* σ^E^ and *C. glutamicum* σ^H^ which are supposed to interact with these nucleotides (Lane and Darst, 2006; Campagne et al., 2014) (shown in bold in Figure 5).

**FIGURE 6:**
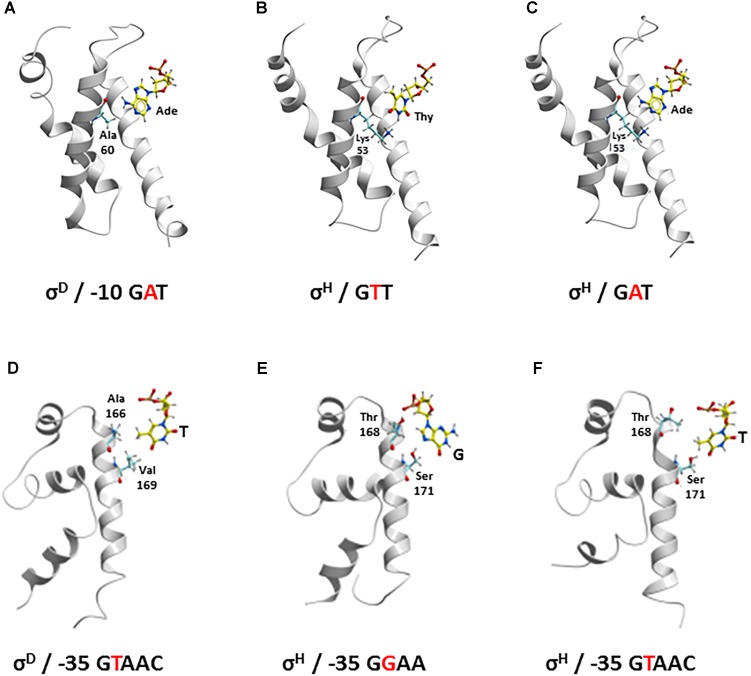
Recognition of the nucleotide at second position of –10 element (yellow represents carbon; red, oxygen; blue, nitrogen; white, hydrogen, Ade, adenine; Thy, thymine) **(A–C)** by Ala 60 in σ^D^ and Lys 53 in σ^H^ (cyan, carbon) and at second position of –35 element (yellow, carbon) **(D–F)** by Ala 166 and Val 169 in σ^D^ and Thr 168 and Ser 171 in σ^H^ (cyan, carbon). –10: **(A)** σ^D^ and σ^D^-dependent promoter; **(B)** σ^H^ and σ^H^-dependent promoter; **(C)** σ^H^ and σ^D^-dependent promoter –35: **(D)** σ^D^ and σ^D^-dependent promoter; **(E)** σ^H^ and σ^H^-dependent promoter; **(F)** σ^H^ and σ^D^-dependent promoter.

As for the -10 element, there is no X-ray or cryo-electron microscopy structure of the RNAP complex with any of the group IV (ECF) sigma factors described, and a model of the complete DNA transcription bubble is not yet available. The key nucleotide at the second position of the *C. glutamicum* -10 elements (i.e., A in σ^D^ and T in σ^H^) is in close contact with the surface of the σ subunit in our homology models based on the *E. coli* σ^E^ crystal structure 4LUP (Figure 6). According to these models, the presence of Ala 60 in σ^D^ and Lys 53 in σ^H^ seems to be crucial for the recognition of the nucleotide at the second position of the -10 element. Ala 60 with the minimal side chain creates space for a larger adenine at the second position in the -10 element at σ^D^ (Figure 6A). In contrast, Lys 53 with a larger side chain fills the free space made by a smaller base, thymine, at the second position of the -10 consensus element at σ^H^ (Figure 6B).

As for the -35 element, template and non-template DNA strands clearly form a duplex. There is no potential conformational polymorphism that could appear with the -10 element, where the transcription bubble opens. Therefore, our homology models of σ^D^ and σ^H^ in complex with the respective -35 elements can be considered highly plausible. To verify the stability and reliability of these homology models, we introduced a water envelope into the models and carried out 50-ns MD simulations. It was found, that the hydrophobic interactions of the methyl group of T at the second position of the σ^D^-consensus of the -35 element with the side chains of Val 169 and Ala 166 in σ^D^ are crucial for its recognition (Figure 6D). Conversely, a hydrogen bond with the side chain of Ser 171 is most important for the recognition of G at the second position of the σ^H^-consensus of the -35 element (Figure 6E). The hydrogen bond between the hydroxyl group of Thr 168 and the phosphate group of the sugar-phosphate DNA backbone provides another stabilizing interaction.

We used the established models to investigate why σ^H^ is, to some degree, capable of recognizing σ^D^-controlled promoters.

#### -10 Element

The larger side chain of Lys 53 in σ^H^ must interact with the larger adenine at the second position of the GAT consensus instead of the small side chain of Ala 60 in σ^D^, if we consider the recognition of the -10 GAT motif of a σ^D^-controlled promoter by σ^H^. This is possible since the Lys 53 side chain is highly flexible and therefore probably does not represent a major barrier to the larger adenine (Figure 6C). To test whether Ala 60 in σ^D^ and Lys 53 in σ^H^ play key roles in recognizing the -10 element in σ^D^-controlled promoters, we designed the mutant σ^H^Lys-53-Ala for *in vivo* analysis.

#### -35 Element

With the -35 element within the σ^D^-dependent promoter, thymine at the second position of the σ^D^-promoter consensus (GTAAC), whose methyl group interacts with the side chains of the hydrophobic AAs Val 169 and Ala 166 in σ^D^ (Figure 6D), interacts with Thr 168 and Ser 171 in σ^H^ (Figure 6F). To assess these interactions in complex (σ^H^/-35 element of σ^D^-dependent promoter) in detail, we again performed an extensive 50-ns MD simulation. It was found that the side chain of Thr 168 in σ^H^ can rotate, and its methyl group then interacts with the methyl group of the thymine within GTAAC, which undoubtedly leads to a hydrophobic stabilization. Moreover, the hydroxyl group from the Ser 171 side chain forms a hydrogen bond with the oxygen atom of the thymine. To test whether the replacement of the AA sequence 168-ThrValMetSerArg-172 with AlaValArgValAla from σ^D^ can improve the recognition of σ^D^-controlled promoters by σ^H^, we designed the mutant σ^H^168-AlaValArgValAla-172 for the *in vivo* analysis.

### Activity of σ^H^-σ^D^ Mutants With the σ^D^-Controlled Promoters

**FIGURE 7:**
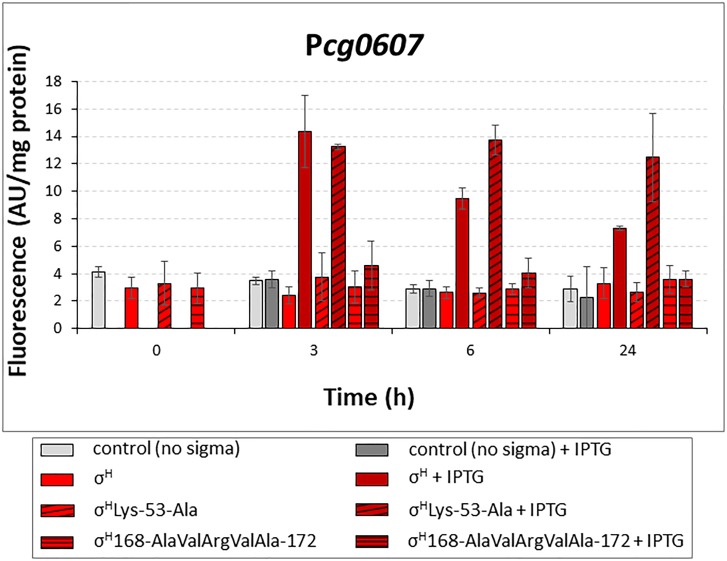
Determination of promoter activity in *C. glutamicum* WT with overexpressed *sigH* or its mutants σ^H^Lys-53-Ala and σ^H^168-AlaValArgValAla-172 by using two-plasmid assay. The *C. glutamicum* strains carried the vector pEC-XT99A overexpressing *sigH* (open bars), σ^H^Lys-53-Ala (cross-hatched bars) or σ^H^168-AlaValArgValAla-172 (horizontally hatched bars) after IPTG addition and the promoter-test vector pEPR1 with the target promoter P*cg0607*. The strain with the empty expression vector pEC-XT99A was used as a control (gray bars). The promoter activity was measured as the Gfpuv fluorescence intensity of the cell extracts. Fluorescence of cultures without IPTG is shown as light bars; fluorescence of cultures with IPTG induction is shown as dark bars. AU, arbitrary units. The standard deviations of three biological replicates are depicted by error bars.

Most of the σ^D^-controlled promoters were to a lesser extent also activated by σ^H^, although the highly conserved sequences of the σ^H^-specific promoters (-35 GGAA^T^/_C_, -10 GTT) differ from those of the identified σ^D^-specific promoters (-35 GTAAC, -10 GAT). Computer modeling showed that σ^H^ can bind to the key regions of promoter DNA in a similar manner as σ^D^ to the analogous elements. Based on the results of the sequence similarity analysis and computer modeling, we designed mutations to make σ^H^ more σ^D^-like and possibly more efficient at transcription from σ^D^-dependent promoters. We introduced the mutations into the *sigH* gene cloned in the expression vector pEC-XT99A, which resulted in the production of the mutant proteins σ^H^Lys-53-Ala or σ^H^168-AlaValArgValAla-172. These mutant genes were used in the two-plasmid assays with the promoters which exhibited the highest activity with σ^H^: P*cg0607*, P*lpps*, P*cmt2*, and P*rsdA*. We assumed that the overproduced σ^H^Lys-53-Ala and σ^H^168-AlaValArgValAla-172 would trigger higher promoter activity in two-plasmid assays with the chosen σ^D^-dependent promoters than σ^H^. This assumption was only confirmed with σ^H^Lys-53-Ala and P*cg0607* (Figure 7). This effect was particularly apparent at T24, when promoter activity with σ^H^Lys-53-Ala was 1.7-fold higher than that with σ^H^. This higher activity compared to other promoters could be explained by the unparalleled presence of the GGAA sequence in the -35 region of P*cg0607*, which is a consensus sequence of -35 elements in σ^H^-dependent promoters. This result indicated that Lys 53 in σ^H^ and Ala 53 in σ^H^Lys-53-Ala (and Ala 60 in σ^D^) most likely interact with the -10 promoter elements.

Contrary to the expectation, σ^H^168-AlaValArgValAla-172 recognized Pcg0607 less efficiently. To assess how the mutant σ^H^168-AlaValArgValAla-172 could interact with the σ^D^-controlled promoters, we analyzed the homology model with this mutant σ factor in more detail. Using a 50-ns MD simulation, we found that the complex of σ^H^168-AlaValArgValAla-172 with the -35 element of the σ^D^-controlled promoter is probably not stable. This was something of a surprise given that the native σ^H^ was able to efficiently bind σ^D^-controlled promoter DNA in previous MD simulations. A detailed analysis of the MD trajectories showed that this instability appeared to be due to the absence of Arg at the final position of the AA sequence AlaValArgValAla (ThrValMetSerArg in σ^H^). The positively charged Arg 172 side chain of σ^H^ was found to create a significant stabilizing interaction (a salt bridge) with the negatively charged backbone of promoter DNA. This hypothesis, which might explain the low efficiency of the mutant σ^H^168-AlaValArgValAla-172, may be tested in the future by creating additional mutations suggested by the *in silico* analysis.

### Multiple Overlapping Promoters Upstream of the σ^D^-Controlled Genes

**FIGURE 8:**
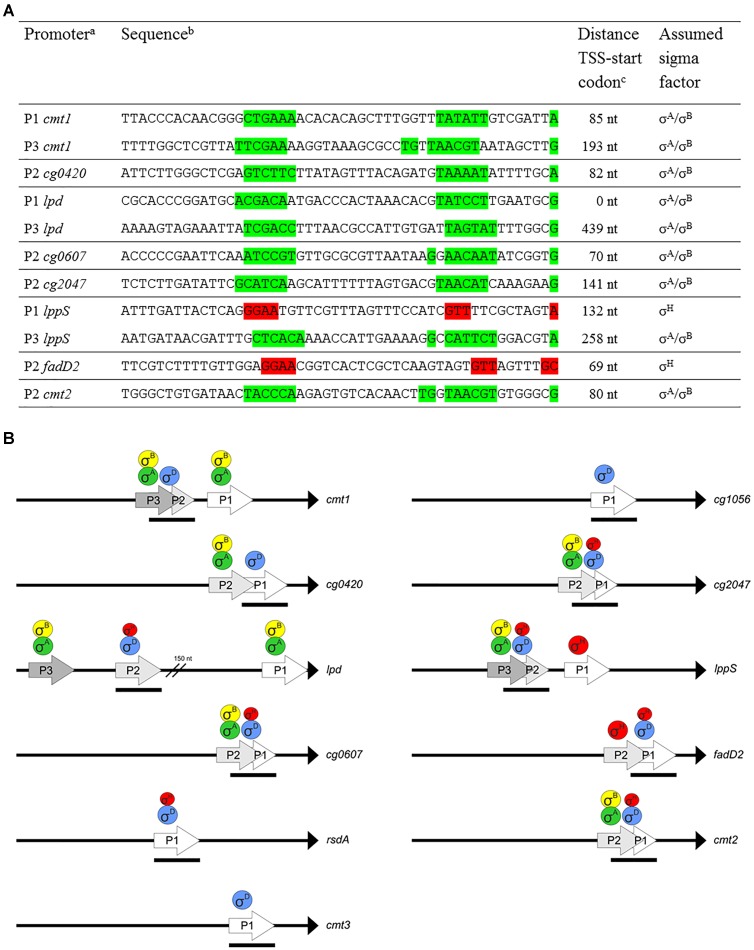
Multiple promoters within upstream region of σ^D^-dependent genes. **(A)** Sequences of putative σ^A^/σ^B^- or σ^H^-dependent promoters within upstream region of σ^D^-dependent genes. The promoters identified closest to the translation start codon were designated P1. The suggested –35 and –10 (or extended –10) promoter elements and the respective TSS are highlighted in green (σ^A^/σ^B^-dependent) or red (σ^H^-dependent). TSS were detected by RNA-seq specific for transcription start sites. **(B)** Schematic representation of upstream regions of the genes with multiple promoters. To complete the studied promoter set, the fragments only carrying the σ^D^-dependent promoters P*rsdA*, P*cmt3*, and P*cg1056* are also included. The proposed sigma factors regulating transcription from the individual promoters are shown above. Transcriptional starts are at the tips of the arrows. The length and positions of the bars below the arrows depict approximately the length and positions of the promoter-carrying DNA fragments (70 nt) used for both *in vitro* and *in vivo* assays. Translation initiation points are shown as black triangles.

In addition to the TSS ascribed to the σ^D^-controlled promoters (Table 2), start sites of transcription driven from different overlapping or nearby promoters were detected by RNA-seq for 8 of the 11 analyzed genes. We suggest that most of these promoters are activated by σ^A^ and/or σ^B^, and two are activated by σ^H^, according to the upstream sequences (Figure 8A, in green and red, respectively). The putative housekeeping promoters are always located upstream of the σ^D^-dependent promoters if there is a single additional σ^A^- and/or σ^B^-dependent promoter (upstream of the *cg0420*, *cg0607*, *cg2047*, and *cmt2* genes). Promoters which we consider to be σ^H^-dependent according to the sequences of the putative -35 (GGAA) and -10 (GTT) elements were found upstream of *lppS* and *fadD2* genes. The detection of all TSS in the upstream regions of the σ^D^-dependent genes was important to avoid effects of transcription started from the additional promoters. The position of the cloned promoter fragments for *in vivo* and *in vitro* assays could thus be chosen in such a way, that only the activity of the supposed σ^D^-controlled promoters was determined. The organization of the multiple promoters within the upstream regions of the 8 genes is shown in Figure 8B. In the case of *fadD2*, the studied σ^D^-dependent P1*fadD2* and the additional σ^H^-dependent P2*fadD2* partially overlap and the activity of the additional σ^H^-specific promoter might thus contribute to the overall σ^H^-dependent activity of the fragment. However, such activity was not detected by *in vitro* transcription. *In vivo*, the σ^H^-dependent activity was similarly low as in other cases. The σ^A^- and/or σ^B^-dependent promoters P3*cmt1*, P2*cg0420*, P2*cg0607*, P2*cg2047*, P3*lppS*, and P2*cmt2* (Figures 8A,B) were found to partially (mostly by the -10 region) reside on the 70-nt fragments with the analyzed σ^D^-dependent promoters. However, their possible activity most probably did not interfere with the *in vivo* measurements of the effects of σ^D^ and σ^H^ overexpression.

### Phenol as a Stressor

We have tested a number of stress conditions (heat shock, SDS, penicillin G, glycine, mitomycin C, phenol and limitation by glucose) to detect a slower growth of the *C. glutamicum* Δ*sigD* strain or to induce transcription from P*sigD* or from the promoters found to be σ^D^-dependent (data not shown). Out of the conditions tested, only phenol exhibited a stronger inhibitory effect on the growth of the Δ*sigD* strain carrying the vector pEC-XT99A than on the growth of the analogous WT strain. We found that growth rate of *C. glutamicum* WT culture in 2xYT medium after the addition of 7.5 mM phenol decreased from 0.88 to 0.46 h^-1^ (1.9-fold), whereas the growth of the *C. glutamicum* Δ*sigD* decreased from 0.68 h^-1^ to 0.22 (3.1-fold). A difference in the effect of phenol was not significant in plasmidless strains.

To analyze the transcriptional response of *C. glutamicum* to phenol stress, we performed RNA-seq using the RNA isolated from the *C. glutamicum* WT cells grown in minimal medium with glucose (2%) or phenol (3.4 mM). Differential gene expression analysis including normalization, was performed using the whole transcriptome data and Bioconductor package DESeq2 (Love et al., 2014) included in the software ReadXplorer v2.2 (Hilker et al., 2014). The evaluation of the differential RNA-seq data was performed using an adjusted *p*-value cut-off of *P* ≤ 0.01 and a signal intensity ratio (*m*-value) cut-off of ≥1 or ≤-1. According to the results (Supplementary Tables S3, S4), five genes previously found to be σ^D^-dependent were more highly expressed on phenol than on glucose: *cg0607, lppS, rsdA, lpd*, and *cg2047* (Supplementary Table S4). The respective TSS identified by RNA-seq of 5′-enriched primary transcripts using the cultures grown on phenol were identical with those found using the *sigD* overexpressing strain.

The effect of phenol on the activity of the promoters P*cg0607*, P*lppS*, P*rsdA*, P*lpd*, and P*cg2047* was tested *in vivo* with the two-plasmid system. The strains carrying *sigD* or *sigH* inserted in pEC-XT99A and the promoters in pEPR1 were cultivated in minimal medium with glucose (2%) or phenol (3.4 mM). The promoter activity was measured in the extract of the cells before the addition of IPTG and at the end of exponential growth phase.

**FIGURE 9:**
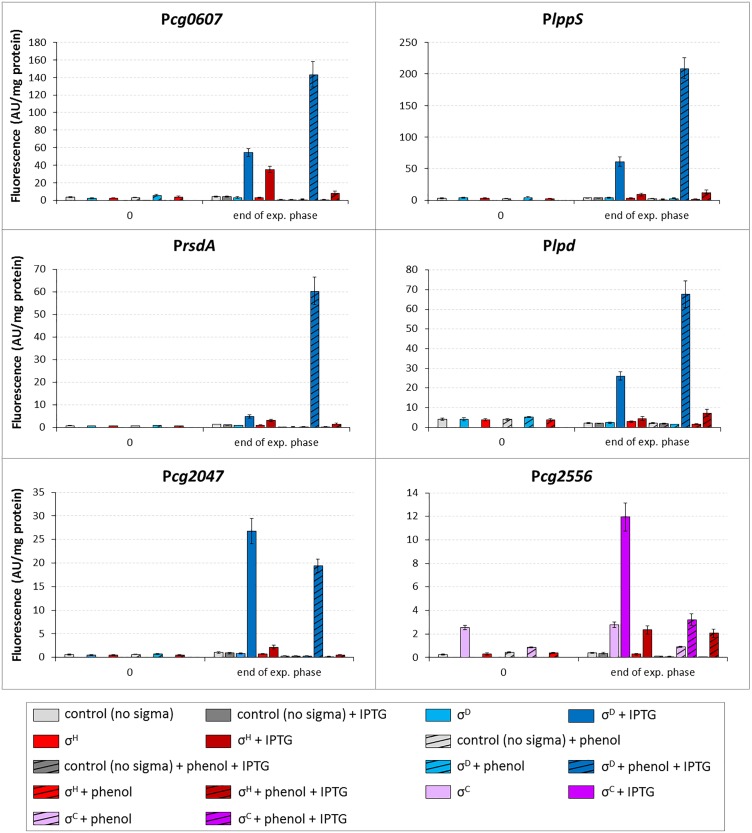
Effect of phenol on activity of σ^D^-dependent promoters. The *C. glutamicum* strains carried the vector pEC-XT99A overexpressing *sigH* (red bars), *sigD* (blue bars) or *sigC* (purple bars) after the addition of IPTG (added at OD_600_ = 1; dark bars) and the promoter-test vector pEPR1 constructs carrying the tested promoters (above the graphs). The strain overexpressing *sigC* and harboring pEPR1 with the σ^C^-dependent promoter P*cg2556* (Dostálová et al., 2017), which is not activated by phenol, was used as a control (purple bars). Phenol (3.4 mM) was present in media from the beginning of cultivation (hatched bars). Plasmids carrying the pEPR1 construct and empty pEC-XT99A were used as controls (gray bars). The samples were taken at OD_600_ = 1 (0) and at the end of exponential growth phase. AU, arbitrary units. The standard deviations of three biological replicates are depicted by error bars.

The activity of four promoters (P*cg0607*, P*lppS*, P*rsdA*, *and* P*lpd*) in the strains with overexpressed *sigD* was clearly higher after cultivation with phenol (Figure 9). The activity of the σ^C^-dependent P*cg2556* used as a control was much lower in the medium with phenol under *sigC* overexpression conditions. The overexpression of *sigH* in the presence of phenol only resulted in a small increase in the P*lpd* activity. These results confirmed the data from RNA-seq (with the exception of P*cg2047* which was induced on phenol according to RNA-seq, but not in the two-plasmid assay). This is one of a few examples of the stress response to the action of phenolic compounds regulated by the ECF sigma factors in *C. glutamicum* (Chen et al., 2016, 2017). The phenol stress response mediated by ECF sigma factors will be further studied in *C. glutamicum* on the basis of the large amount of data produced by RNA-seq.

## Discussion

We recently began to study the function of *C. glutamicum* σ^D^ and its regulon for the first time (Taniguchi et al., 2017). The ECF sigma factor σ^D^ was found to be crucial for the envelope stress response, the synthesis of the mycomembrane and formation of the cell wall (Taniguchi et al., 2017; Toyoda and Inui, 2018). The genes of the σ^D^ regulon are involved in the synthesis of mycolic acids, the modification of peptidoglycan and other cell envelope-related functions. A subgroup of these genes which control mycolate metabolism are specific to the Mycolata group of bacteria, which include *Corynebacterium, Mycobacterium*, *Rhodococcus*, and *Nocardia* as the most important genera.

Sigma factors exhibiting a high level of similarity to *C. glutamicum* σ^D^ have been found in many bacteria. The highest level of AA identity with *C. glutamicum* σ^D^ was detected in σ^D^ proteins deduced from the respective genes in species closely related to *C. glutamicum* [*C. deserti* (94%), *C. callunae* (93%) and *C. efficiens* (90%)]. A lower level of identity (70–79%) was found with σ^D^ proteins from most of the phylogenetically more distant *Corynebacterium* species. The identity of *C. glutamicum* σ^D^ with σ^D^ proteins from other bacteria of the Mycolata group (e.g., genera *Mycobacterium*, *Rhodococcus*, and *Nocardia*) exhibited a range of 54–57%. All bacteria with a σ^D^ identity level higher than 45% (e.g., streptomycetes) belong to *Actinobacteria*. However, the functions of the genes of the σ^D^ regulons in these related bacteria are partially different. In addition to mycolate synthesis, the *M. tuberculosis*σ^D^ regulon is involved in virulence, lipid metabolism and gene expression under starvation conditions (Raman et al., 2004; Calamita et al., 2005).

A comparison of the promoter sequences of the putative σ^D^-dependent genes among various bacteria was limited by the fact that either homologous genes were only found in a small number of *Corynebacterium* species (e.g., the genes *cg0420* and *cg2047* only occur in the genomes of three *C. glutamicum* strains) or no consensus sequences suggested for σ^D^-dependent promoters were identified upstream of the homologous genes in the vast majority of other *Corynebacterium* species. Conservation of the putative σ^D^-dependent promoter sequences among various bacteria was only found in the genes *cmt1* and *cmt2*, encoding trehalose corynomycolyl transferases.

Almost total conservation (37/38) of the -10 and -35 sequences (GTAAC - 15 nt – CTCGAT) in the P*rsdA* promoter was found in 38 *Corynebacterium* species encoding the anti-sigma factor RsdA. In other Actinobacteria (genera *Rhodococcus*, *Nocardia*, and *Mycobacterium*), a putative σ^D^-dependent promoter is not present upstream of the *rsdA* gene but upstream of the *sigD* gene. Such an arrangement indicates that the *sigD-rsdA* operon is autoregulated by σ^D^ in these bacteria, in contrast to *C. glutamicum*, where σ^D^ seems to only control *rsdA* gene expression but not its own synthesis. Only two vegetative promoters were localized upstream of the *C. glutamicum* ATCC 13032 *sigD* gene (Pfeifer-Sancar et al., 2013).

We showed that σ^D^ played a dominant role in the transcription from all promoters tested in this study, however, low σ^H^ activity with most of these promoters was detected by the *in vivo* two-plasmid assay. This activity of the promoters was not observed when using *in vitro* transcription (with one exception). The promoter P*cg0607* was an exception and its efficiency documented by *in vitro* transcription was even higher with σ^H^ than with σ^D^ (Figure 2). The reason for this is probably that the -35 sequence GGAA within P*cg0607* is identical to the consensus of the -35 element of σ^H^-controlled promoters. Similarly, the -10 element GTT, typical for σ^H^-controlled promoters, was found in P*lpd*. Its activity with σ^H^ *in vivo* was also higher than that of other promoters (Figure 3). We can consider P*cg0607* and P*lpd* to be natural σ^D^/σ^H^-dependent hybrid promoters. However, it is apparent that the promoter efficiencies determined *in vivo* and *in vitro* do not correlate perfectly.

SigH plays a prominent role in the sigma regulatory network. Promoters controlled by σ^H^ were found to drive the transcription of *sigA* (Toyoda and Inui, 2015), *sigB* (Dostálová et al., 2017) and *sigM* (Nakunst et al., 2007). SigH, which also controls the expression of several genes encoding transcriptional regulators (*clgR, sufR*, and *hspR*), is therefore considered to be a candidate for a global regulatory molecule (Schröder and Tauch, 2010). Overlap of the recognition specificities of σ^H^ and σ^E^ was described for some promoters (Šilar et al., 2016). In this study, an unexpected overlap of σ^H^ and σ^D^ in recognizing σ^D^-dependent promoters has been proven.

To prove that the promoters are recognized directly with RNAP+σ^H^, we measured the activities of P*cmt2*, P*lppS*, and P*rsdA* with σ^H^ in the Δ*sigD* strain. All three promoters were found to be active (Figure 4). The transcription driven by σ^H^ was apparently stronger in the absence of σ^D^ in the cell, probably because there was no competition with σ^D^. Upstream regions of *cmt2* and *rsdA* carried no potential σ^H^-specific promoter and the possible σ^H^-specific promoter P1*lppS* was not included within the tested 70-nt P2*lppS* fragment (distance between TSS1 and TSS2 was 93 nt). Thus, it was apparently σ^H^, which drove the detected activity of the σ^D^-dependent promoter P2*lppS* in the Δ*sigD* mutant. Thus, σ^H^ was able to partially substitute for missing σ^D^.

We have investigated the σ^D^-dependent genes by different methods in *C. glutamicum* ATCC 13032 than those used in *C. glutamicum* R (Toyoda and Inui, 2018). In our study, differential gene expression analysis based on the obtained RNA-seq data of the whole transcriptome libraries (Illumina TruSeq stranded mRNA libraries) and RNA-seq of the 5′-enriched primary transcripts of *C. glutamicum* ATCC 13032 overexpressing *sigD* enabled us to not only discover the σ^D^-regulated genes, but also precisely identify the TSS of the genes studied. Moreover, individual σ^D^-dependent promoters were analyzed using *in vitro* transcription and *in vivo* two-plasmid assay. The *in vitro* assay revealed that P*cg0607* can directly drive transcription with RNAP+σ^H^ (Figure 2). Two σ^D^-dependent genes of *C. glutamicum* ATCC 13032, *cg0420* and *cg2047* (encoding glycosyltransferase and a secreted protein, respectively), are not present on the *C. glutamicum* R chromosome. Another gene, *lpd* was not found to be σ^D^-dependent in *C. glutamicum* R. The promoter P2*lpd* was localized 360 nt upstream of the translation initiation codon. There is a possibility that a potential small RNA, which was detected by RNA-seq, is transcribed from this promoter. P2*lpd* has a GTT sequence in the -10 region which is the consensus of σ^H^-dependent promoters. The *lpd* gene (encoding dihydrolipoamide dehydrogenase) is known to play a role in the oxidative stress response in bacteria (Krisko et al., 2014). In *M. tuberculosis lpd* contributes to survival against host-generated reactive oxygen species (Daly et al., 2007). The activity of P2*lpd* was found to increase with *sigD* and, to a lesser extent, with *sigH* overexpression in the presence of phenol in *C. glutamicum* (Figure 9).

Based on the *in silico* analysis, the *sigH* mutants resulting in the production of the mutant proteins σ^H^Lys-53-Ala or σ^H^168-AlaValArgValAla-172 were constructed. The mutation Lys-53-Ala within the -10 sequence-binding region of σ^H^ improved the σ^H^-controlled activity of P*cg0607*, which is in agreement with the suggested crucial role of Ala in this position in recognizing the -10 element of σ^D^-controlled promoters. The low promoter activity with σ^H^ mutant carrying 168-AlaValArgValAla-172 was probably due to the missing Arg 172, which may establish important stabilizing salt bridges with the sugar-phosphate backbone of DNA according to the *in silico* homology modeling.

Differential gene expression analysis of the transcriptome of *C. glutamicum* ATCC 13032 grown with or without phenol revealed that some σ^D^-dependent genes (*cg0607*, *lppS, rsdA*, and *cg2047*) were higher transcribed under the phenol treatment. The promoters P*cg0607*, P*lppS*, P*rsd* and in addition P*lpd*, were also found to be induced by phenol in minimal medium by two-plasmid analysis (Figure 9). In *C. glutamicum* R, differential gene expression analyzed by microarrays showed that the σ^D^-dependent genes play a role in the response to the lysozyme treatment (Toyoda and Inui, 2018). The changes in gene expression seem to be induced by the cell envelope stress exerted by the action of phenol or lysozyme.

Phenol in complete medium did not significantly slow the growth of the Δ*sigD* strain compared to the WT strain. However, when the replication of a single or even two plasmids placed a burden on the cell growth, these plasmid-harboring Δ*sigD* strains grew slower or did not grow at all. This extremely poor growth of Δ*sigD* strain with two plasmids was probably due to deficiencies in the cell envelope synthesis in combination with detrimental effects of supplemented antibiotics (Km and Tc) necessary for plasmid maintenance.

The function of σ^D^-dependent genes and the organization of their transcription suggest that these genes play an important role not only in stress response, but also in cell homeostasis and in building a cell envelope during rapid growth under optimum conditions. Under optimal growth conditions, the transcription of the genes involved in corynomycolate synthesis is probably driven from additional σ^A^- and/or σ^B^-dependent promoters or σ^H^-dependent promoters, which we detected upstream of many σ^D^-dependent genes (Figure 8A). The transcription of σ^D^-dependent genes from multiple promoters controlled by other sigma factors and the transcription from the σ^D^-dependent promoters by σ^H^ may partially ensure the expression of these genes if the σ^D^ function is damaged or eliminated. However, these promoters are probably not strong enough to enable the cell to cope with the combined stress conditions if σ^D^ is missing. The low σ^H^-triggered activity of the largely σ^D^-controlled promoters, which is probably induced by modulation of the σ^H^ recognition specificity under particular physiological conditions, may contribute to fine-tuning transcription under various stresses, especially under the cell envelope stress.

Several genes of the *M. tuberculosis* σ^D^ regulon were found to be active in the stationary growth phase (Raman et al., 2004). Gfpuv fluorescence, which should reflect the activity of *C. glutamicum* σ^D^-dependent promoters in our study, increased during cultivation and was found to be maximal at T24 (Figure 3). Interestingly, activity of the same promoters driven by σ^H^ exhibited the opposite trend. It therefore seems that σ^H^ may partially substitute for σ^D^ activity during the exponential phase.

The possible global regulator σ^H^ has been proven to play a crucial role in the hierarchy of σ factors, since it drives the transcription of at least three of them (σ^A^, σ^B^, and σ^M^). Moreover, the regulatory overlaps of σ^H^ with transcriptional regulators(Toyoda and Inui, 2015) and with several sigma factors including σ^D^ seem to control a number of physiological functions in *C. glutamicum* including heat, SOS, oxidative, chemical and cell surface stress responses. The accumulated evidence indicates that overlaps between the regulons of *C. glutamicum* ECF sigma factors are rather a common regulatory strategy how to cope with complex environmental stresses than an unusual exception.

## Author Contributions

MP and JK conceived the project and led the studies performed in Prague and Bielefeld, respectively. HD, TB, LR, and VŠ carried out the most experiments. TB and HD carried out RNA-seq and processed the data. JH did the *in vitro* transcription assays. JN performed the sequence analyses. IB carried out *in silico* analyses. All authors analyzed the results. MP and JN drafted the initial manuscript. All authors contributed to writing the final manuscript, reading, and approving the submitted version.

## Conflict of Interest Statement

The authors declare that the research was conducted in the absence of any commercial or financial relationships that could be construed as a potential conflict of interest.

## Supplementary Material

Table S1 Oligonucleotides used.

Table S2 List of identified TSSs of SigD-upregulated genes and the respective sequences as detected by RNA-seq of transcriptome of *C. glutamicum* with overexpressed *sigD* cultivated on glucose.

Table S3 List of identified TSSs of and the respective sequences as detected by RNA-seq of transcriptome of *C. glutamicum* WT cultivated on glucose.

Table S4 List of identified SigD-dependent TSSs of SigD-upregulated genes and the respective sequences as detected by RNA-seq of transcriptome of *C. glutamicum* WT cultivated on phenol.
