# Supplementary material for: Overlap of Promoter Recognition Specificity of Stress Response Sigma Factors SigD and SigH in Corynebacterium glutamicum ATCC 13032
Source: Front Microbiol. 2019 Jan 9;9:3287. doi: 10.3389/fmicb.2018.03287 (PMC6338062; doi:10.3389/fmicb.2018.03287)
Supplement: Table S1 — Oligonucleotides used. [file Table_1.DOCX]

**Table S1** Oligonucleotides used

| Primer | Sequence^a^ | Use |
| --- | --- | --- |
| PCG1056770F  PCG1056770R  PCMT2770F  PCMT2770R  PFADD2770F  PFADD2770R  P1AD770F  P1AD770R  PCG2047P770F  PCG2047P770R  PLPPS770F  PLPPS770R  PCG0441UP770F  PCG0441UP770R  PCG0607P770F  PCG0607P770R  PCG0420770F  PCG0420770R  PCMT3770F  PCMT3770R  PCMT1P770F  PCMT1P770R  CLPP1P770F  CLPP1P770R  PCG1056PEPRF  PCG1056PEPRR  PCMT2PEPRF  PCMT2PEPRR  PFADD2PEPRF  PFADD2PEPRR  P1ADPEPRF  P1ADPEPRR  PCG2047F  PCG2047R  PLPPSPEPRF  PLPPSPEPRR  PCG0441UPF  PCG0441UPR  PCG0607F  PCG0607R  SIGHmutK-AF  SIGHmutK-AR  SIGHmutTMSR-ARVAF  SIGHmutTMSR-ARVAR | *AATT****C***AGCAGTAATTTGTTTTGACGACGCAGTAACGCAATCGGGGATTGTGGTCGATTCTTTAAGCAAG***A***  ***AGCTT***CTTGCTTAAAGAATCGACCACAATCCCCGATTGCGTTACTGCGTCGTCAAAACAAATTACTGCT***G***  ***A****ATT****C***GGCTGTGATAACTACCCAAGAGTGTCACAACTTGGTAACGTGTGGGCGGAAAAACAAGATAGGCATCGAGAG*A*  ***AGCTT***CTCTCGATGCCTATCTTGTTTTTCCGCCCACACGTTACCAAGTTGTGACACTCTTGGGTAGTTATCACAGCC***G***  ***AATTC***GAACGGTCACTCGCTCAAGTAGTGTTAGTTTGCAAAAGTAATAAAATGTTCATCTTTGTCGATGGTCACAATAG***A***  ***AGCTT***CTATTGTGACCATCGACAAAGATGAACATTTTATTACTTTTGCAAACTAACACTACTTGAGCGAGTGACCGTTC***G***  *AA****TT****C*TTTGCGCTGGTCAGCGATGGAAGTAACAGAGTTAGGGAACTTCTCGATCTACTGAGTGAA*A*  ***AG****CTT*TTCACTCAGTAGATCGAGAAGTTCCCTAACTCTGTTACTTCCATCGCTGACCAGCGCAAA*G*  ***A****AT****TC***TCGCATCAAGCATTTTTTAGTGACGTAACATCAAAGAAGTATTCACTGATGTAAGTAGTGGACTGAGC***A***  *A****GCTT***GCTCAGTCCACTACTTACATCAGTGAATACTTCTTTGATGTTACGTCACTAAAAAATGCTTGATGCGA***G***  ***A****ATT****C***GCTCACAAAACCATTGAAAAGGCATTCTGGACGTAACGCTCCGGCATCTACAAGGGATGATCAAAATAGCTAC*A*  ***AG****C****T****T*GTAGCTATTTTGATCATCCCTTGTAGATGCCGGAGCGTTACGTCCAGAATGCCTTTTCAAT GGTTT  TGTGAGC***G***  ***AATTC***CGACTTGCAAATAGTTCATTTCGGCAGAGTGCTAACGGTTAGGCACTATTTTCCGTTAGTTCTTTTGTAGTC***A***  *A****G****C****TT***GACTACAAAAGAACTAACGGAAAATAGTGCCTAACCGTTAGCACTCTGCCGAAATGAACTATTTGCAAGTCG***G***  ***AATTC***AATTCAAATCCGTGTTGCGCGTTAATAAGGAACAATATCGGTGTGATTCGCGATATATTAATCAGCTT***A***  *A****GCTT***AAGCTGATTAATATATCGCGAATCACACCGATATTGTTCCTTATTAACGCGCAACACGGATTTGAATT***G***  ***AATTC***AAATATTTTGCATCTGAGCAGTTAGAAACGGTATGTCGGTAGTAACCGATACGATTTATTGAAGCT***A***  ***AGCTT***AGCTTCAATAAATCGTATCGGTTACTACCGACATACCGTTTCTAACTGCTCAGATGCAAAATATTT***G***  ***AATTC***ATGCACCGGGGGAAACTATGGGGATTTTGGGGGAGGTTGTTACAAAACCATACGTCTGTGAAGATATGACGAGTGC***A***  ***AGCTT***GCACTCGTCATATCTTCACAGACGTATGGTTTTGTAACAACCTCCCCCAAAATCCCCATAGTTTCCCCCGGTGCAT***G***  *A****A****TTC*GAAAAGGTAAAGCGCCTGTTAACGTAATAGCTTGAAATATAGATGTAAATTA*A*  *A****G****CTT*TAATTTACATCTATATTTCAAGCTATTACGTTAACAGGCGCTTTACCTTTTC*G*  ***AATTC***AGCGAACAGAGGCGGTTTCATGGAAATACGCGGGTAGTC**C**GGTGACATTGAACCAAATGA***A***  ***AGCTT***TCATTTGGTTCAATGTCACC**G**GACTACCCGCGTATTTCCATGAAACCGCCTCTGTTCGCT***G***  ***G***AGCAGTAATTTGTTTTGACGACGCAGTAACGCAATCGGGGATTGTGGTCGATTCTTTAAGCAAGGGTAATGTCGAAAC***G***  ***G****A****TCC***GTTTCGACATTACCCTTGCTTAAAGAATCGACCACAATCCCCGATTGCGTTACTGCGTCGTCAAAACAAATTACTGCT***CTGCA***  *G*GCTGTGATAACTACCCAAGAGTGTCACAACTTGGTAACGTGTGGGCGGAAAAACAAGATAGGCATCGAGAGGTATCAGCGGTCCA***G***  ***GATCC***TGGACCGCTGATACCTCTCGATGCCTATCTTGTTTTTCCGCCCACACGTTACCAAGTTGTGACACTCTTGGGTAGTTATCACAGC*C****TGCA***  *G*GAACGGTCACTCGCTCAAGTAGTGTTAGTTTGCAAAAGTAATAAAATGTTCATCTTTGTCGATGGTCACAATAG***G***  ***G****AT****CC***CTATTGTGACCATCGACAAAGATGAACATTTTATTACTTTTGCAAACTAACACTACTTGAGCGAGTGACCGTTC*CT****G****CA*  TATG*CT****GCA****G*CTCGTATTGGAGG  CCAA***G****GAT****CC***CTCTCAAGATAAGAATGT  ***G***TCGCATCAAGCATTTTTTAGTGACGTAACATCAAAGAAGTATTCACTGATGTAAGTAGTGGACTGAGC*G*  ***G****ATC****C***GCTCAGTCCACTACTTACATCAGTGAATACTTCTTTGATGTTACGTCACTAAAAAATGCTTGATGCGA***C****T****GCA***  *G*CTCACAAAACCATTGAAAAGGCATTCTGGACGTAACGCTCCGGCATCTACAAGGGATGATCAAAATAGCTACATGAGGAAATGTT*G*  ***G****A****TC****C*AACATTTCCTCATGTAGCTATTTTGATCATCCCTTGTAGATGCCGGAGCGTTACGTCCAGAATGCCTTTTCAATGGTTTTGTGAG*C****TGCA***  *G*CGACTTGCAAATAGTTCATTTCGGCAGAGTGCTAACGGTTAGGCACTATTTTCCGTTAGTTCTTTTGTAGTC***G***  *G****ATCC***GACTACAAAAGAACTAACGGAAAATAGTGCCTAACCGTTAGCACTCTGCCGAAATGAACTATTTGCAAGTCG*CT****GCA***  *G*AATTCAAATCCGTGTTGCGCGTTAATAAGGAACAATATCGGTGTGATTCGCGATATATTAATCAGCTT*G*  ***G****A****TC****C*AAGCTGATTAATATATCGCGAATCACACCGATATTGTTCCTTATTAACGCGCAACACGGATTTGAATT*C****T****G****CA***  CACCTATATC**gcg**GCGTACCAGG  TCTTGCACGAGATCTTCC  **cgtcgca**CTCCATCGTGGAAGAAAACAG  **cg**cac**agc**TCCGAGTGGAACGTCCAT | P*cg1056* cloning in pRLG770  P*cg1056* cloning in pRLG770  P*cmt2* cloning in pRLG770  P*cmt2* cloning in pRLG770  P*fadD2* cloning in pRLG770  P*fadD2* cloning in pRLG770  *PrsdA* cloning in pRLG770  *PrsdA* cloning in pRLG770  P*cg2047* cloning in pRLG770  P*cg2047* cloning in pRLG770  *PlppS* cloning in pRLG770  *PlppS* cloning in pRLG770  P*cg0441* cloning in pRLG770  P*cg0441* cloning in pRLG770  P*cg0607* cloning in pRLG770  P*cg0607* cloning in pRLG770  P*cg0420* cloning in pRLG770  P*cg0420* cloning in  pRLG770  P*cmt3* cloning in pRLG770  P*cmt3* cloning in pRLG770  P*cmt1* cloning in pRLG770 P*cmt1*cloning in pRLG770  P*clpP1* cloning in  pRLG770  P*clpP1* cloning in  pRLG770  P*cg1056* cloning in pEPR1  P*cg1056* cloning in pRLG770  P*cmt2* cloning in pEPR1  P*cmt2* cloning in pEPR1  P*fadD2* cloning in pEPR1  P*fadD2* cloning in  pEPR1  *PrsdA* cloning in  pEPR1  *PrsdA* cloning in pEPR1  P*cg2047* cloning in pEPR1  P*cg2047* cloning in pEPR1  *PlppS* cloning in pEPR1  *PlppS* cloning in pEPR1  P*cg0441* cloning in pEPR1  P*cg0441* cloning in pEPR1  P*cg0607* cloning in pEPR1  P*cg0607* cloning in pEPR1  *sigH* mutagenesis  *sigH* mutagenesis  *sigH* mutagenesis  *sigH* mutagenesis |

^a^ sites for restriction enzymes are in italics, mismatched nucleotides are in bold
